# Genetic variation at the microRNA binding site of CAV1 gene is associated with lung cancer susceptibility

**DOI:** 10.18632/oncotarget.21687

**Published:** 2017-10-09

**Authors:** Xue Fang, Xuelian Li, Zhihua Yin, Lingzi Xia, Xiaowei Quan, Yuxia Zhao, Baosen Zhou

**Affiliations:** ^1^ Department of Epidemiology, School of Public Health, China Medical University, Shenyang, China; ^2^ Liaoning Provincial Department of Education, Key Laboratory of Cancer Etiology and Prevention, China Medical University, Liaoning, China; ^3^ Department of Radiotherapy, The Fourth Affiliated Hospital of China Medical University, Shenyang, China; ^4^ Department of Epidemiology, School of Public Health, Shenyang Medical College, Shenyang, China

**Keywords:** single nucleotide polymorphism, lung cancer, CAV1, microRNA

## Abstract

Single nucleotide polymorphism (SNP) may influence the genesis and development of cancer in a variety of ways depending on their location. Here we conducted a study in Chinese female non-smokers to investigate the relationship between rs1049337, rs926198 and the risk or survival of lung cancer. Further, we explored whether rs1049337 could alter the binding affinity between the mRNA of CAV1 and the corresponding microRNAs. Finally, we evaluated the relationship between expression level of CAV1 and prognosis of lung cancer. The results showed that the rs1049337-C allele and rs926198-C allele were the protective alleles of lung cancer risk. Haplotype analysis indicated that the C-C haplotype (constructed by rs1049337 and rs926198) was a protective haplotype for lung cancer risk. The result of luciferase reporter assay showed that rs1049337 can affect the binding affinity of CAV1 mRNA to the corresponding microRNAs both in A549 cell line and H1299 cell line. Compared with C allele, T allele had a relatively decreased luciferase activity. Compared with paired normal adjacent tissue or normal lung tissue, lung cancer tissue showed a relatively low level of CAV1. Refer to those patients at early stage of lung cancer, the expression level of CAV1 in patients at late stage of lung cancer was relatively low. In conclusion, the results indicated that rs1049337, it's a SNP located at 3′UTR region of CAV1 may affect lung cancer risk by altering the binding affinity between the mRNA of CAV1 and the corresponding microRNAs.

## INTRODUCTION

Lung cancer is the most frequently diagnosed lethal malignancies in the world. As the poor 5-year survival rate, lung cancer has become the leading cause of death among females in more developed countries and males in the whole world. As is known to all, smoking is regarded as a common risk factor of lung cancer. Global cancer statistics state that 15% of men and 53% of women with lung cancer were not caused by smoking [1]. Smoking rates in Chinese women are lower than some European countries, but the incidence of lung cancer in China is relatively high [2]. This suggested us that some other factors may be involved in lung cancer in Chinese female population.

Caveolae is a plasma membrane invagination and juxtamembrane vesicles, which play a role in the macromolecular vesicle transportand can be regarded as a kind of lipid raft. Due to the ubiquitous tissue distribution of caveolae, it has been confirmed that caveolae involved a large number of cellular functions. Caveolin-1 (CAV1) protein is a principal structural component of caveolae and found in the cytosolic surface of caveolae, which called caveolar coat protein. In mammalian cells, CAV1 protein is quite small and highly conserved which can recruit many signaling molecules to caveloae and regulate their activity [3–5].

CAV1 has been found to be involved in a variety of cancer-associated biological processes, including cell migration and metastasis, cell transformation, angiogenesis, multidrug resistance [6–9]. CAV1 is an interesting gene for cancer as it may play different roles in different types of cancer at different stages. No consistent results regarding whether CAV1 is an oncogene or tumor suppressor gene. Most studies suggest that CAV1 may play a positive role in the early stages of cancer onset. In advanced tumors, CAV1 is frequently associated with poor prognosis and drug resistance [10].

Single nucleotide polymorphism (SNP) is a kind of single nucleotide change widely distributed in the human genome. There is increasing evidence suggesting that SNPs may play a critical role in the genesis and development of cancer [11–14]. The relationship between SNPs in CAV1 and cancer risk has not been extensively studied. In the present study, we performed a case-control study to assess the relationship between SNPs in CAV1 and the risk or survival of lung cancer in Chinese female non-smokers.

## RESULTS

### Basic characteristics of subjects

Table 1 listed the basic information of the subjects. A total of 923 subjects were included in the present study, including 468 lung cancer cases and 455 age-matched controls. All subjects were Chinese female nonsmokers, and there was no significant difference between cases and controls with regard to age (*P*=0.861). Among cases, there were 402 non-small cell lung cancer (NSCLC) and 66 small cell lung cancer (SCLC). Among the cases with NSCLC, there were 322 cases with adenocarcinoma, 66 cases with squamous cell carcinoma and 14 cases with a variety of different rare pathologies.

**Table 1:** Characteristics of included subjects

Variables	Cases (%)	Controls (%)	*P* value
Females	468	455	
Mean age (years)	56.16±11.73	56.3±11.46	0.861
Histological			
Adenocarcinoma	322 (68.8%)		
Squamous cell carcinoma	66 (14.1%)		
Small cell lung cancer	66 (14.1%)		
Others^a^	14 (3.0%)		
Stage^b^			
I	10(2.3)		
II	109(24.7)		
III	267(60.5)		
IV	55(12.5)		

^a^Including adenosquamous carcinoma, mixed cell carcinoma, large cell carcinoma and undifferentiated carcinoma.

^b^There are missing values.

### The effect of SNP on the risk of lung cancer

First we evaluated the relationship between the two SNPs and lung cancer risk. The results were summarized in Table 2. Genotype distributions of rs1049337 and rs926198 in control group were all in accordance with the HWE (*P*>0.05). Subjects carrying rs1049337 CC genotypes showed a decreased risk of lung cancer compared to the subjects carrying homozygous TT genotype (adjusted OR=0.691, 95%CI=0.482-0.990, *p*=0.044). Recessive model showed that take TT+TC genotype as reference group, CC genotype was the protective genotype of lung cancer risk (adjusted OR=0.688, 95%CI=0.510-0.929, *P*=0.015). Further analyses were carried on by allele comparison, the result indicated that compared with T allele, C allele was a protective allele of lung cancer risk (adjusted OR=0.829, 95%CI=0.691-0.995, *P*=0.045). For rs926198, taking rs926198-TT genotype as reference group, TC genotype showed a decreased risk of lung cancer (adjusted OR=0.552, 95%CI=0.367-0.830, *P*=0.004). Dominant model and additive model of rs926198 also showed statistically significant results (adjusted OR=0.557, 95%CI=0.372-0.834, *P*=0.005, adjusted OR=0.583, 95%CI=0.396-0.860, *P*=0.006, respectively).

**Table 2:** Genetic polymorphisms and risk of lung cancer

	Genotype	Lung cancer cases (%)	Controls (%)	*P* of HWE	Adjusted OR ^a^	95% CI	*P*
Rs1049337	TT	133 (28.4)	118(25.9)	0.061	Ref		
	TC	234 (50.0)	207(45.5)		1.006	0.738,1.373	0.968
	CC	101 (21.6)	130(28.6)		0.691	0.482,0.990	0.044^*^
Dominant model					0.882	0.659,1.179	0.395
Recessive model					0.688	0.510,0.929	0.015^*^
Additive model	C allele				0.829	0.691,0.995	0.045^*^
Rs926198	TT	425(90.8)	385(84.6)	0.506	Ref		
	TC	42(9.0)	69(15.2)		0.552	0.367,0.830	0.004^*^
	CC	1(0.2)	1(0.2)		0.910	0.056,14.674	0.947
Dominant model					0.557	0.372,0.834	0.005^*^
Recessive model					1.007	0.251,4.042	0.992
Additive mode	C allele				0.583	0.396,0.860	0.006^*^

^a^Adjusted for age, ORs and 95% CIs were calculated by logistic regression. ^*^ *P*<0.05.

The results of stratified analysis were showed in Table 3 and Supplementary Table 1. In NSCLC cases and controls, rs926198 showed remarkable results. Compared with subjects carrying TT genotype, those whom carrying TC genotype had an adjusted OR of 0.551 (95%CI=0.359-0.846, *P*=0.006) for developing NSCLC. Dominant model and additive model of rs926198 also showed significant results.

**Table 3:** Genetic polymorphisms and risk of NSCLC

	Genotype	Controls (%)	NSCLC (%)	Adjusted OR ^a^	95% CI	*P*	Adenocarcinoma (%)	Adjusted OR ^a^	95% CI	*P*
Rs1049337	TT	118(25.9)	114(28.4)	Ref			93(28.9)	Ref		
	TC	207(45.5)	201(50.0)	1.008	0.984,1.011	0.962	167(51.9)	1.028	0.731,1.444	0.875
	CC	130(28.6)	87(21.6)	0.694	0.477,1.009	0.056	62(19.3)	0.605	0.402,0.909	0.015^*^
Dominant model				0.884	0.653,1.195	0.421		0.862	0.626,1.187	0.363
Recessive model				0.692	0.506,0.946	0.021^*^		0.594	0.421,0.839	0.003^*^
Additive model	C allele			0.830	0.686,1.003	0.054		0.782	0.638,0.957	0.017^*^
Rs926198	TT	385(84.6)	365(90.8)	Ref			286(88.8)	Ref		
	TC	69(15.2)	36(9.0)	0.551	0.359,0.846	0.006^*^	35(10.9)	0.683	0.442,1.055	0.085
	CC	1(0.2)	1(0.2)	1.081	0.067,17.459	0.956	1(0.3)	1.317	0.0781,21.289	0.846
Dominant model				0.559	0.366,0.853	0.007^*^		0.692	0.450,1.964	0.093
Recessive model				1.176	0.073,18.967	0.909		1.406	0.087,22.699	0.810
Additive model	C allele			0.588	0.392,0.882	0.010^*^		0.720	0.477,1.086	0.117

^a^Adjusted for age, ORs and 95% CIs were calculated by logistic regression. ^*^ *P*<0.05.

The results of analysis in patients with lung adenocarcinoma and controls indicated that recessive model and additive model of rs1049337 showed decreased lung adenocarcinoma risk (adjusted OR=0.594, 95%CI=0.421-0.839, *P*=0.003, adjusted OR=0.782, 95%CI=0.638-0.957, *P*=0.017, respectively). Compared with rs1049337-TT genotype, CC genotype was a protective genotype of lung adenocarcinoma (adjusted OR=0.605, 95%CI=0.402-0.909, *P*=0.015). In squamous cell carcinoma, rs926198 showed significant results, as the small sample size of squamous cell carcinoma patients, the results need to be verified in the future.

### Haplotype analyses of the SNPs and lung cancer risk

**Figure 1:**
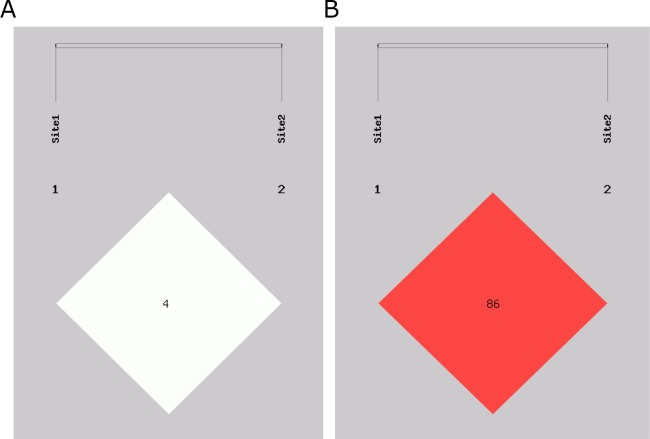
Linkage disequilibrium structure of two SNPs in CAV1 Site1 is rs104937. Site2 is 926198. **(A)** D’ linkage map of two SNPs in CAV1. **(B)** R2 linkage map of two SNPs in CAV1.

We analyzed the relationship between haplotype and lung cancer risk. The haplotypes were constructed by rs1049337 and rs926198 (Table 4), four common haplotypes were observed. The linkage Disequilibrium (LD) plots are shown in Figure 1. C-C haplotye was a protective haplotype in lung cancer and NSCLC (OR=0.495, 95%CI=0.329-0.744, *P*<0.001; OR=0.510, 95%CI=0.334-0.780, *P*=0.001; respectively). Compared with the combination of all the other haplotypes, T-T haplotype showed an increased risk of adenocarcinoma (OR=1.261, 95%CI=1.030-1.545, *P*=0.025).

**Table 4:** Haplotypes and the risk of lung cancer (rs1049337-rs926198)

Haplotype^a^	Controls (%)	Lung cancer			NSCLC			Adenocarcinoma		
		N (%)	OR (95%CI)	*P*	N (%)	OR (95%CI)	*P*	N (%)	OR (95%CI)	*P*
CC	71(7.8)	37(4.0)	0.495(0.329-0.744)	<0.001^*^	33(4.1)	0.510(0.334-0.780)	0.001^*^	32(5.0)	0.627(0.408-0.963)	0.032^*^
CT	396(43.5)	399(42.6)	0.975(0.811-1.172)	0.787	342(42.5)	0.971(0.801-1.176)	0.762	259(40.2)	0.883(0.719-1.084)	0.234
TC	0(--)	7(0.7)	--	--	5(0.6)	--	--	5(0.7)	--	--
TT	443(48.7)	493(52.7)	1.193(0.993-1.432)	0.059	424(52.7)	1.192(0.986-1.442)	0.070	348(54.1)	1.261(1.030-1.545)	0.025^*^

^a^Frequency of haplotypes <3% were excluded from the final analysis. ^*^ *P*<0.05

### The effect of SNPs on the overall survival of lung cancer

The results of the relationship between genetic polymorphisms and survival time were summarized in Table 5, Supplementary Table 2 and Supplementary Figure 1. We have not observed any statistically significant association between rs1049337, rs926198 and lung cancer survival time.

**Table 5:** Distribution of genotypes and survival time of lung cancer patients

SNP	Genotype	Lung cancer (%)	MST (mon)	Log-rank *P*	Adjusted HR ^a^	95% CI
Rs1049337	TT	97(27.6)	24.5		Ref	
	TC	175(49.9)	24.5	0.889	1.012	0.775,1.321
	CC	79(22.5)	25.8		0.952	0.688,1.316
Dominant model		254(72.4)	24.9	0.952	0.991	0.770,1.275
Recessive model		272(77.5)	24.5	0.636	0.935	0.710,1.231
Rs926198	TT	317(90.3)	28.3		Ref	
	TC+CC	34(9.7)	24.4	0.247	0.791	0.535,1.168

^a^ Adjusted for age, HRs and 95% CIs were calculated by Cox regression.

### Luciferase reporter assay

Supplementary Table 3 listed the predication results of microRNAs those may bind to CAV1 mRNA around rs1049337. Considering the predication score, finally hsa-miR-612 was selected to verify its relationship with rs1049337.

**Figure 2:**
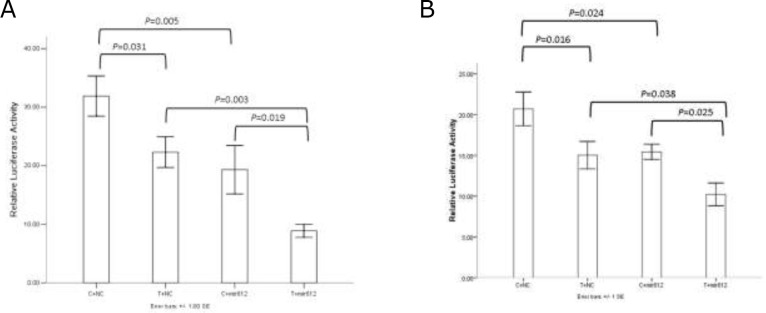
The results of dual luciferase reporter assay **(A)** Results in A549 cell line. **(B)** Results in H1299 cell line.

The result showed that rs1049337 can affect the binding affinity of CAV1 mRNA to some microRNAs (Figure 2). In the A549 cell line and the H1299 cell line, we got essentially identical results. Compared with C allele, T allele had a relatively decreased relative luciferase activity. When miR-612 mimics existed in cell, T allele had a lower expression level of CAV1 compared with C allele. Compared with mimics negative control, the relative luciferase activity of vectors containing C allele and T allele was significantly decreased when miR-612 mimics existed. The results indicated that rs1049337 can affect the binding affinity of CAV1 mRNA to the corresponding microRNA, these microRNAs include miR-612.

### The association of CAV1 expression level and lung cancer

Finally, we screened four datasets in GEO database which were suitable for our study. The results of the analysis of the relationship between CAV1 expression level and NSCLC were listed in Table 6. We found that compared with paired normal adjacent tissue or normal lung tissue, lung cancer tissue showed a relatively low level of CAV1 mRNA (*P*<0.001, *P*<0.001, respectively). In three datasets (GSE19804, GSE27716 and GSE40275), we found that referring to those patients at early stage of lung cancer, the expression level of CAV1 in patients at late stage was relatively low (*P*=0.007, *P*=0.005, *P*=0.001, respectively). In GSE27716, a borderline statistical difference was found in the comparison of CAV1 mRNA levels in noninvasive lung adenocarcinoma patients and invasive lung adenocarcinoma patients (*P*=0.08).

**Table 6:** The association of CAV1 expression level and lung cancer

GEO dataset	Variable	N	Median^a^	*P*
GSE19804	Tissue			
	Paired normal adjacent tissue	60	13.192	<0.001^*^
	NSCLC tissue	60	10.728	
	Stage			
	I	34	11.093	0.007^*^
	II / III / IV	26	10.054	
GSE27716	Invasive			
	Noninvasive lung adenocarcinoma specimen	17	8.717	0.080
	Invasive lung adenocarcinoma specimen	23	7.450	
	Stage			
	Ia	23	8.355	0.005^*^
	Ib / II / III	17	7.437	
GSE29013	Histological			
	Squamous cell carcinoma	25	8.097	0.001^*^
	Adenocarcinoma	30	7.398	
	Stage			
	I	24	7.852	0.519
	II / III	31	7.689	
	Chemotherapy			
	Yes	34	7.598	0.100
	No	21	7.954	
GSE40275	Subjects			
	Normal lung sample	43	9.577	<0.001^*^
	NSCLC sample	18	7.963	
	SCLC sample	23	5.883	
	Gender			
	Female	36	9.464	<0.001^*^
	Male	48	7.963	
	Stage			
	I	20	7.772	0.001^*^
	II / III	21	5.795	

^a^Expression level of CAV1. ^*^ *P*<0.05.

## DISCUSSION

The role of CAV1 in cancer is oncogene or tumor suppressor gene hasn't been agreed yet, and the role of CAV1 in lung cancer has rarely been studied [3, 4, 7]. We conducted a study to explore the relationship between CAV1 gene polymorphism, expression level and lung cancer. We found that the CAV1-rs1049337, CAV1-rs926198 may affect the susceptibility of lung cancer in the Chinese female non-smokers. Further experiments revealed that compared with rs1049337-T allele, the increased relative luciferase activity was found in C allele. It was proved that the different allele of rs1049337 could affect the binding affinity of CAV1 to the corresponding microRNA. We also found that CAV1 expression may play a positive role in lung cancer.

MicroRNA is a class of single stranded noncoding RNA which is considered to regulate large number of genes at post transcriptional level. The regulatory effect of microRNA on gene expression is achieved by microRNA seed region complementary base pairing to the 3′UTR region of the target gene [15, 16]. Any nucleotide change in 3′UTR region of target gene can affect the affinity of target gene and microRNA, and further influence the expression level of target gene. Rs1049337 is located at 3′UTR region of CAV1 gene. Our study found that compared with rs1049337-T allele, C allele was a protective allele for risk of lung cancer. Further investigation demonstrated that rs1049337 can affect the binding affinity of CAV1 mRNA to the corresponding microRNAs. Finally, we found that CAV1 expression may play a positive role in lung cancer. As far as we know, there are very few studies on the relationship between rs1049337 and disease. A study on the relationship between genetic variation in CAV1 and long-term pancreas transplant function found that rs1049337 is a tag SNP of CAV1, but there's no significant correlation between long-term graft function, long-term pancreas function and rs1049337 was observed in donors and recipients respectively [17]. The MuTHER study was found that rs1049337 is an expression Quantitative Trait Loci (eQTL), and another study based on the MuTHER study revealed that rs1049337 is an eQTL with an age-by-genotype interaction effect associated to GWAS hits [18, 19]. Although we have studied the mechanism of how rs1049337 affects lung cancer, considering racial differences and the credibility of a single study, multicenter replication studies still need to be done.

Rs926198 is located at intron region of CAV1. Although we haven't figured out how does intron SNPs play a role in disease, a large number of genome-wide association study (GWAS) have confirmed that intron SNPs are associated with a variety of diseases in a quite robust *P* value [20–22]. In our study, we found that compared with rs926198-T allele, C allele was a protective allele for lung cancer risk. To the best of our knowledge, it's the first study to explore the relationship between rs926198 and cancer. Previous studies indicated that rs926198 is associated with metabolic syndrome, fasting insulin levels, insulin resistance, hyperinsulinemic, hypertension and systemic sclerosis [23–26]. After further study, Rene Baudrand et al. found that in inadipose tissues, rs926198 minor allele is associated with a lower expression level of CAV1 [24]. So it is necessary to further explore the mechanism of how rs926198 plays a role in the disease.

CAV1 is localized to 7q31.2, closed to D7S522 locus which is a fragile site that is frequently absent in cancer and is thought to be a suspected tumor suppressor locus [27]. Indeed, CAV1 is considered to play a positive role in some cancer studies, but the results of some other studies were exactly the opposite. Whether *in vitro* or in animal or in cancer patients, CAV1 is a tumor promoter gene or tumor suppressor gene that has not been consistent. Breast cancer is the most widely studied cancer with CAV1. One study found that compared with normal mammary epithelial counterparts, CAV1 showed a significant decrease in breast cancer cell, and loss of CAV1 expression was found in P53 deficient cells [28]. CAV1 can inhibit the growth and metastasis of breast cancer [29]. The molecular mechanism study revealed that in human breast cancer-associated fibroblasts, down regulated CAV1 expression play a key role in maintaining the abnormal phenotype [30, 31]. Breast cancer patients with loss CAV1 expression in stromal cells were associated with shorter survival time, increased risk of early tumor recurrence, higher recurrence rate and metastasis, higher CAV1 expression in tumor cells were associated with poor survival [32–37]. Other studies have suggested that CAV1 plays a negative role in breast cancer. In human breast cancer cell, CAV1 accumulates at invadopodia and its knockdown inhibits invadopodia formation [38]. CAV1 is thought to be a potential causative factor of trastuzumab resistance generation in breast cancer cells [39]. In prostate cancer cell, CAV1 is thought to promote metastatic activities, proangiogenic activities, promote tumor progression and promotes lymphangiogenesis [40–43]. In prostate cancer stroma, loss of CAV1 associated with reduced relapse-free survival [44]. The positive expression of CAV1 was more observed in high grade urothelial carcinoma and bladder cancer patients [45, 46]. In human transitional bladder cancer cell with drug resistance, the expression level of CAV1 was found to be elevated [47]. But one study has found that compared with normal tissue, CAV1 had a decreased expression in bladder cancer tissues [48]. The expression of CAV1 in colon carcinoma was up-regulated or down-regulated, the results of different studies have been quite different [49–51]. In Apc (min/+) mice with deficiency of CAV1, a promoted colorectal tumorigenesis was observed [52]. A meta analysis showed that overexpression of CAV1 was associated with a better overall survival in gastric cancer patients [53].

In CAV1 null (CAV-1 -/-) mouse model, hypercellularity with thickened alveolar septa was observed in lung parenchyma, disorganize and multilayer was observed in alveolar wall [54, 55]. In lung cancer cell lines, there is controversy about whether CAV1 is expressed. Compared with lung epithelial cell, one study showed that the expression of CAV1 was reduced in lung adenocarcinoma cell lines [56]. In another study, CAV1 was found to be reduced in small cell lung cancer cell lines, but in 76% of NSCLC cell lines, it is still expressed [57]. In lung cancer patients, compared with the adjacent tissues or normal lung tissue, the expression of CAV1 was down-regulated in cancer tissues [58–62]. But CAV1 was more frequently expressed in NSCLC patients those with drug resistance and poor prognosis [60, 63–65]. In our study, CAV1 may be considered to play a positive role in the genesis and development of lung cancer. Further study on the relationship between CAV1 and cancer is urgently needed

## MATERIALS AND METHODS

### Study subject

This study is approved by the ethics committee of China Medical University. Each participant signed an informed consent before participating in the study. All of the participants were unrelated Chinese Han population and non-smoking women. Patients with lung cancer were recruited from the First Affiliated Hospital of China Medical University and Liaoning Cancer Hospital& Institute. All of the patients were newly detected and had not received any radiotherapy or chemotherapy. Over the same period, controls were recruited from the health examination center of the same hospital and frequency-matched to cases in age.

### Data collection and SNP genotyping

Clinical information of patients was collected from patients’ medical records. By face-to-face interviews, we obtained the personal information from each subject. The data was recorded age, status of smoking, family history of cancer, etc. Subjects who had smoked less than 100 cigarettes in their lifetime were defined as non-smokers. Each subject was collected 10ml of venous blood. DNA was extracted from whole blood using standard Phenol-chloroform Method and was stored at -20°C for subsequent experiments. We carried out the follow up by telephone among the patients once every three months. SNPs were genotyped by using the Illumina 660W SNP microarray (Illumina Inc., San Diego, CA).

### Cell culture and transfection

We purchased A549 and H1299 cell line from Shanghai Institute of Biochemistry and Cell Biology, Chinese Academy of Sciences. A549 and H1299 cell lines were cultured in RPMI-1640 medium (Biological Industries, Israel) supplemented with 10% fetal bovine serum (Biological Industries, Israel), 80U/ml penicilin and 0.08mg/ml streptomycin, followed by incubation with 5% CO_2_ at 37°C. A549 and H1299 cell lines transiently transfected using Opti-MEM (Gibco, USA) and Lipofectamine 3000 (Invitrogen, USA) according to the manufacturer's instructions.

### Luciferase reporter assay

In order to screen out those microRNAs that may binding to 3′UTR region of CAV1 mRNA around rs1049337, we adopted 4 database for microRNA predication, including MirSNP [66], miRNASNP [67], PolymiRTS Database 3.0 [68], SNPinfo web server [69].

MicroRNA mimics were purchased from GenePharma (GenePharma, China). A 602-bp fragment of CAV1 3′UTR containing rs1049337-C or rs1049337-T were amplified and cloned in GV272 vector (GeneChem, China). All reporter plasmid were verified by direct sequencing. Cell were cultured in 24-well plates, transient transfection was performed at the logarithmic phase. 48 hours after transfection, the firefly luciferase and renilla luciferase were quantified by Dual-Luciferase Reporter Assay System (Promega, USA). We performed three independent experiments and each was done in triplicate.

### The association of CAV1 expression level and lung cancer

In order to evaluate whether the expression level of CAV1 can affect the progression and prognosis of lung cancer patients, we systematically searched the GEO database to obtain the NSCLC gene expression profile datasets with prognostic information [70, 71]. Data extraction were using GEO2R web tool in GEO database. If there are multiple probes corresponding to the same gene, the average value of these probes was calculated as the expression level of the gene.

### Statistical analysis

Student's *t*-test (for continuous variable) and chi-squared test (for categorical variables) were used to measure the difference between two groups in the distribution of demographic characteristics. Comparison of continuous variables between three or more groups were used one-way analysis of variance (ANOVA). In control group, the Pearson chi-squared test was adopted to determine whether the genotype frequencies follow Hardy-Weinberg equilibrium (HWE). Unconditional logistic regression model was applied to calculate the odds ratios (ORs) and their 95% confidence intervals (CIs) to evaluate the relationship between SNPs and lung cancer risk with adjustment for age. The haplotypes frequency estimation and the analysis of association between haplotypes and lung cancer risk were using SHEsis online web-server [72, 73]. Statistical analyses were carried out using SPSS 22.0 software (IBM, USA). All *p* value were two sided and *p*<0.05 was considered statistically significant.

## SUPPLEMENTARY MATERIALS FIGURE AND TABLES


