# Supplementary material for: Genetic variation at the microRNA binding site of CAV1 gene is associated with lung cancer susceptibility
Source: Oncotarget. 2017 Oct 9;8(54):92943–54. doi: 10.18632/oncotarget.21687 (PMC5696234; doi:10.18632/oncotarget.21687)
Supplement: Supplementary file 1 [file oncotarget-08-92943-s001.pdf]

Genetic variation at the microRNA binding site of CAV1 gene is associated with lung cancer susceptibility

SUPPLEMENTARY MATERIALS

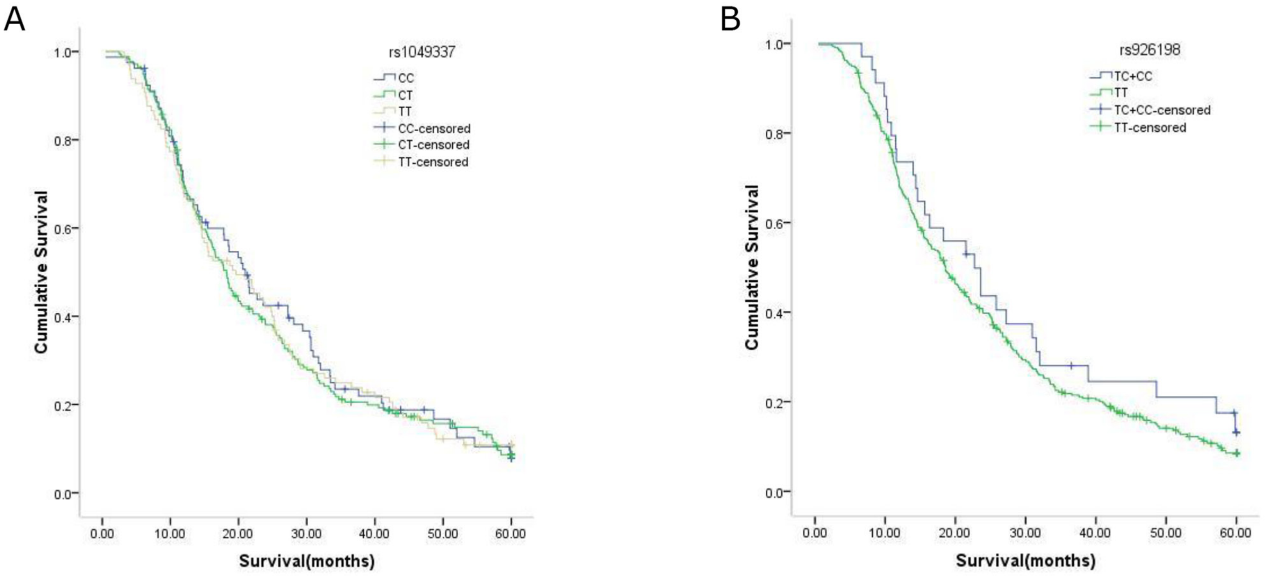

Supplementary Figure 1: Genotypes of rs1049337, rs926198 and its association with NSCLC survival time. (A) Rs1049337. **(B)** rs926198.

Supplementary Table 1: Genetic polymorphisms and risk of lung cancer

	Genotype	Controls (%)	SCLC (%)	Adjusted OR [a]	95% CI	*P*	Squamous cell carcinoma	Adjusted OR [a]	95% CI	*P*
Rs1049337	TT	118(25.9)	19(28.8)	Ref			18(27.3)	Ref		
	TC	207(45.5)	33(50.0)	0.992	0.539,1.824	0.979	29(43.9)	0.916	0.488,1.720	0.783
	CC	130(28.6)	14(21.2)	0.678	0.325,1.416	0.301	19(28.8)	0.974	0.487,1.947	0.940
Dominant model				0.862	0.486,1.529	0.611		0.935	0.523,1.672	0.820
Recessive model				0.662	0.354,1.239	0.197		1.029	0.581,1.824	0.921
Additive model	C allele			0.809	0.560,1.167	0.256		0.986	0.684,1.420	0.938
Rs926198	TT	385(84.6)	6(9.1)	Ref			1(1.5)	Ref		
	TC+CC	70(15.4)	60(90.9)	0.546	0.227,1.313	0.176	65(98.5)	0.085	0.012,0.625	0.015*
Additive mode	C allele			0.559	0.238,1.314	0.182		0.091	0.013,0.660	0.018*

[a]Adjusted for age, ORs and 95% CIs were calculated by logistic regression. * $P<0.05$.

Supplementary Table 2: Distribution of genotypes and survival time of NSCLC

SNP	Genotype	NSCLC (%)	MST (mon)	Log-rank *P*	Adjusted HR [a]	95% CI	Adenocarcinoma (%)	MST (mon)	Log-rank *P*	Adjusted HR [a]	95% CI
Rs1049337	TT	88(28.2)	23.2		Ref		71(29.0)	24.2		Ref	
	TC	153(49.0)	22.7	0.811	1.015	0.764,1.346	123(50.2)	22.9	0.827	1.076	0.782,1.481
	CC	71(22.8)	24.9		0.921	0.656,1.294	51(20.8)	25.4		0.894	0.656,1.445
Dominant model			23.4	0.912	0.980	0.751,1.278		23.6	0.808	1.039	0.769,1.404
Recessive model			22.8	0.526	0.911	0.682,1.217		23.3	0.655	0.924	0.658,1.298
Rs926198	TT	283(90.7)	22.9		Ref		219(89.4)	23.1		Ref	
	TC+CC	29(9.3)	27.5	0.217	0.761	0.496,1.168	26(10.6)	28.7	0.185	0.732	0.461,1.163

[a]Adjusted for age, HRs and 95% CIs were calculated by Cox regression.

Supplementary Table 3: The results of microRNA predication

Database	Prediction results
MirSNP	has-miR-1285-3p, has-miR-3187-5p, has-miR-3609, has-miR-4311, has-miR-4708-5p, has-miR-5189, has-miR-520g, has-miR-520h, has-miR-548ah-5p, has-miR-612
miRNASNP 1.0	has-miR-4311
PolymiRTS Database 3.0	has-miR-6757-5p, has-miR-4311
SNPinfo web serve	has-miR-1231, has-miR-1285, has-miR-518a-5p, has-miR-527, has-miR-612
